# Pseudoerisypèle orbito-palpébral révélant un adénocarcinome gastrique

**DOI:** 10.11604/pamj.2014.18.300.5114

**Published:** 2014-08-14

**Authors:** Rajae Derrar, Rajae Daoudi

**Affiliations:** 1 Université Mohammed V Souissi, Service d'Ophtalmologie A Hôpital des Spécialités CHU Rabat, Maroc

**Keywords:** Pseudoerisypèle orbito-palpébral, adénocarcinome gastrique, tumefaction, Pseudo-erysipelas of the eyelid, gastric adenocarcinoma, swelling

## Image en medicine

Nous présentons le cas d'un patient âgé de 45 ans, présentant une tumefaction pseudo-érysipéloide orbito palpébrale gauche (A) d'apparition rapidement progressive avec extension jugale homolatérale et des placards durs infiltrés au niveau de la région scapulaire (B), le tout évoluant dans un tableau d'amaigrissement non chiffré, d'altération de l’état général et d’épigastralgies rebelles aux antalgiques. Une TDM orbito-cérébrale a démontrée (C) la présence d'un processus tumoral orbitaire et conjonctivo-palpébral infiltrant les muscles oculomoteurs, la graisse orbitaire et les parties molles. L’étude histopathologique d'une biopsie orbitaire conclu à une métastase d'un carcinome indifférencié en cellule à bague à chaton probablement d'origine gastrique, confirmé par une FOGD et une biopsie gastrique. Ces carcinomes sont réputés pour être les tumeurs les plus pourvoyeuses de métastases. Les métastases orbitaires des carcinomes digestifs sont rares, disséminant par voie hématogène et se manifestant parfois sous forme de pseudoérysipèle. Notre patient a reçu une chimiothérapie à base de 5 fluorouracile et de cisplatine complétée par une radiothérapie palliative, il est décédé 3 mois plus tard. L'approche diagnostique des métastases orbitaires reste difficile. Le prognostic reste sombre à cause de la découverte tardive des cancers primitifs impliqués.

**Figure 1:**
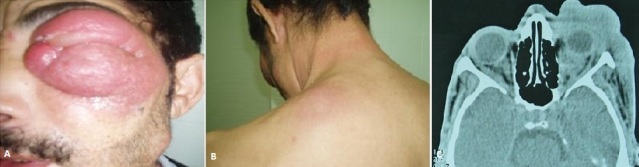
A):tuméfaction pseudo-érysipéloide orbito palpébrale gaucheavec extension jugale homolatérale; (B): des placards durs infiltrés au niveau de la région scapulaire; C) coupes axiales démontrant la présence d'un processus tumoral orbitaire et conjonctivo-palpébral infiltrant les muscles oculomoteurs, la graisse orbitaire et les parties molles

